# Left ventricular assist device implantation in high risk destination therapy patients: an alternative surgical approach

**DOI:** 10.1186/1749-8090-7-21

**Published:** 2012-03-12

**Authors:** Louis E Samuels, Elena Casanova-Ghosh, Roberto Rodriguez, Christopher Droogan

**Affiliations:** 1 Department of Surgery, Division of Cardiothoracic Surgery, Lankenau Medical Center, Wynnewood, PA; 2 Department of Medicine, Division of Cardiology, Lankenau Medical Center, Wynnewood, PA

**Keywords:** Clinical Review

## Abstract

Left Ventricular Assist Device (LVAD) for Destination Therapy (DT) is an established therapy for end stage heart failure patients who are not transplant candidates. Many DT patients requiring LVADs have had prior open heart surgery, the majority of whom had prior sternotomy. In addition, DT patients tend to be older and more likely to have more significant co-morbidities than their Bridge-To-Transplant (BTT) counterparts. As such, placement of an implantable LVAD in DT patients can be technically hazardous and potentially prone to more perioperative complications. The purpose of this report is to describe an alternative implantation approach for the implantation of the Heartmate II™ LVAD in high risk DT patients.

## Background

Left Ventricular Assist Device (LVAD) for Destination Therapy (DT) has been an established therapy for over a decade [1] with the Heartmate VE™ and more recently with the Heartmate II™ (Thoratec Corporation, Pleasonton, CA). The use of this technology for DT has increased as the number of advanced heart failure patients continue to grow and the number of available organs for transplantation has remained essentially unchanged [2]. As such, more patients are being screened for DT-LVAD therapy when deemed ineligible for transplant; a risk strategy tool--the Destination Therapy Risk Score (DTRS)--has been adopted to help determine operative risk [3,4]. However, certain parameters are not taken into consideration in the DTRS, including the presence of prior heart surgery and certain co-morbidities (e.g. COPD, morbid obesity, cerebrovascular and peripheral vascular disease, intractable arrhythmia, cancer, etc.). DTRS values exceeding 16 are considered high risk; DTRS score exceeding 19 are considered very high risk. The purpose of this report is to describe an alternative implantation technique of the Heartmate II LVAD for high risk DT patients that minimizes the potential for surgically related complications.

## Cases

### Case 1

A 71 year old man with end-stage Idiopathic Dilated Cardiomyopathy (IDCM) was admitted with acute decompensated heart failure while on home Inotrope (Milrinone) therapy. His past medical history (PMH) was significant for morbid obesity (BMI = 36), hypertension, hyperlipidemia, diabetes, chronic renal insufficiency, cerebrovascular disease, and obstructive sleep apnea. His past surgical history (PSH) consisted of an ACORN CorCap™ with concomitant mitral valve annuloplasty five years earlier and bi-ventricular ICD-pacemaker. The Destination Therapy Risk Score was calculated at 20.

Preoperative carotid screening revealed high grade stenosis of both internal carotid arteries. Staged carotid endarterectomies were performed, after which a HM II LVAD ™ was implanted. There were no intraoperative complications. Postoperatively, the patient experienced respiratory complications with multiple reintubations, Klebsiella pneumonia, and eventual tracheostomy. In addition, he also experience atrial and ventricular arrhythmias requiring multiple adjustments and changes in anti-arrhythmic therapies. Two months were required to wean from the ventilator; the patient was discharged on postoperative day (POD) 93 and remains alive and well at home six months since discharge.

### Case 2

A 71 year old man with end-stage ischemic cardiomyopathy (ISCM) was admitted with acute decompensated heart failure accompanied by multiple Implantable Cardiac Defibrillator (ICD) shocks. His PMH was significant for chronic renal insufficiency, dyslipidemia, cerebrovascular disease, ventricular arrhythmia, gout, and anemia of chronic disease. The PSH was significant for coronary artery bypass grafting (CABG) fourteen years earlier at which time he suffered a perioperative stroke with subsequent recovery. He also had a single chamber ICD placed three years earlier. An IABP was placed and medical management with Milrinone was instituted prior to the LVAD implant. Significant biochemical and hematologic abnormalities included a BUN of 65 MG/DL, Creatinine 2.2 MG/DL, hematocrit (HCT) 30.2%, PLT count 97 K/UL, and INR 1.5. The Destination Therapy Risk Score was calculated at 20.

Preoperatively, the patient was treated for upper extremity phlebitis and pneumonia. Two weeks were required to optimize his condition for the LVAD procedure including placement of an IABP and Milrinone therapy. No intraoperative problems were encountered. Postoperatively, the patient required surgical exploration of the femoral artery (following IABP removal) including thrombectomy and vascular repair. In addition, he required re-intubation from aspiration pneumonia with subsequent ventilator dependence requiring tracheostomy. Six weeks were required to wean from the ventilator during which time he experienced intermittent, but significant hematuria requiring cystoscopy and fulguration of bleeding vessels in the prostate and bladder. Anti-arrhythmic therapy was also adjusted on multiple occasions to control intermittent ventricular tachycardia. The patient was discharged on POD# 94 and remains alive at a rehabilitation facility two months since discharge.

### Case 3

A cachectic 77 year old man end-stage ISCM was admitted with progressive shortness of breath. His PMH was significant for an acute anterior wall MI twenty-five years earlier and multiple percutaneous coronary interventions over the ensuing years. In addition, he had multiple ablative procedures for ventricular tachycardia (VT) and eventual placement of a biventricular ICD. Over the decades, medical therapy was uptitrated and optimized until a year prior to admission when hypotension required reduction in dosing; additionally, he lost twenty pounds. Socially, he consumes alcohol daily and smoked 3 packs per day for 35 years, having quit at the time of his first MI. His height, weight, and BMI on admission were six feet, 140 pounds, and 1.53 respectively. Intravenous Milrinone therapy was instituted prior to the LVAD implant. The Destination Therapy Risk Score was calculated at 17.

The LVAD was implanted with no intraoperative problems were encountered. Postoperatively, however, the patient experienced bleeding from the chest tubes requiring bedside re-exploration of the left thoracotomy incision. A significant amount of blood was found in the left chest with no identifiable surgical source. Laboratory studies showed a postoperative INR of 3.1 and a Platelet count of 47 K/UL. Evacuation of the hemothorax and blood product transfusion stabilized the situation. The patient developed hepato-renal failure--maximum total bilirubin19.3 mg/DL and creatinine 4.7 mg/DL-- requiring hemodialysis; these biochemical abnormalities resolved within two weeks. He did, though, remain intubated and required tracheostomy for ventilator dependence. Pseudomonas bacteremia was also diagnosed and treated with quinolone based antibiotics. He was eventually stable for discharge on POD # 94. He was discharged to a rehabilitation facility and recently discharged to home.

### Implantation technique

**Figure 1:**
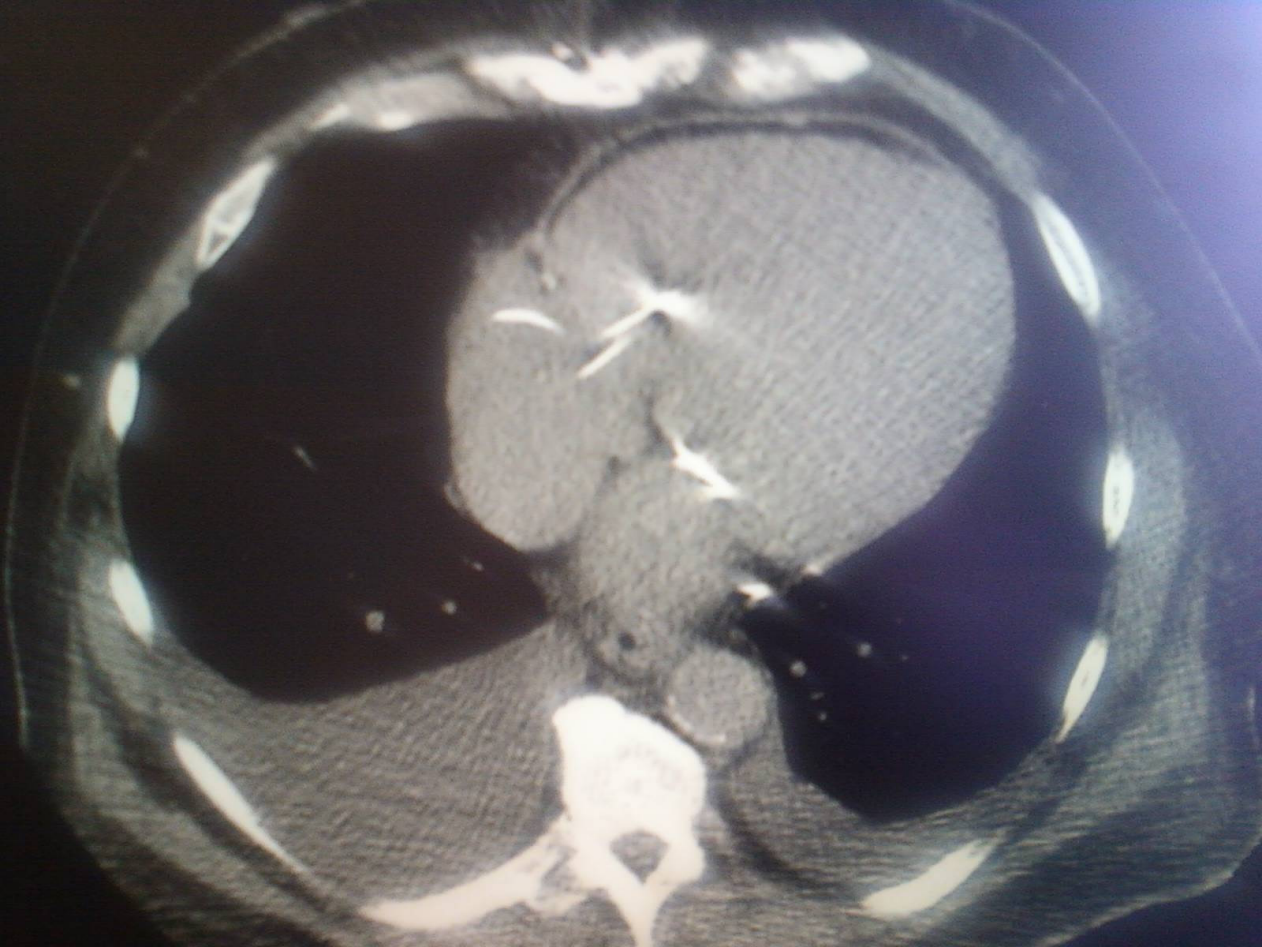
**Preoperative Chest CT Scan**.

**Figure 2:**
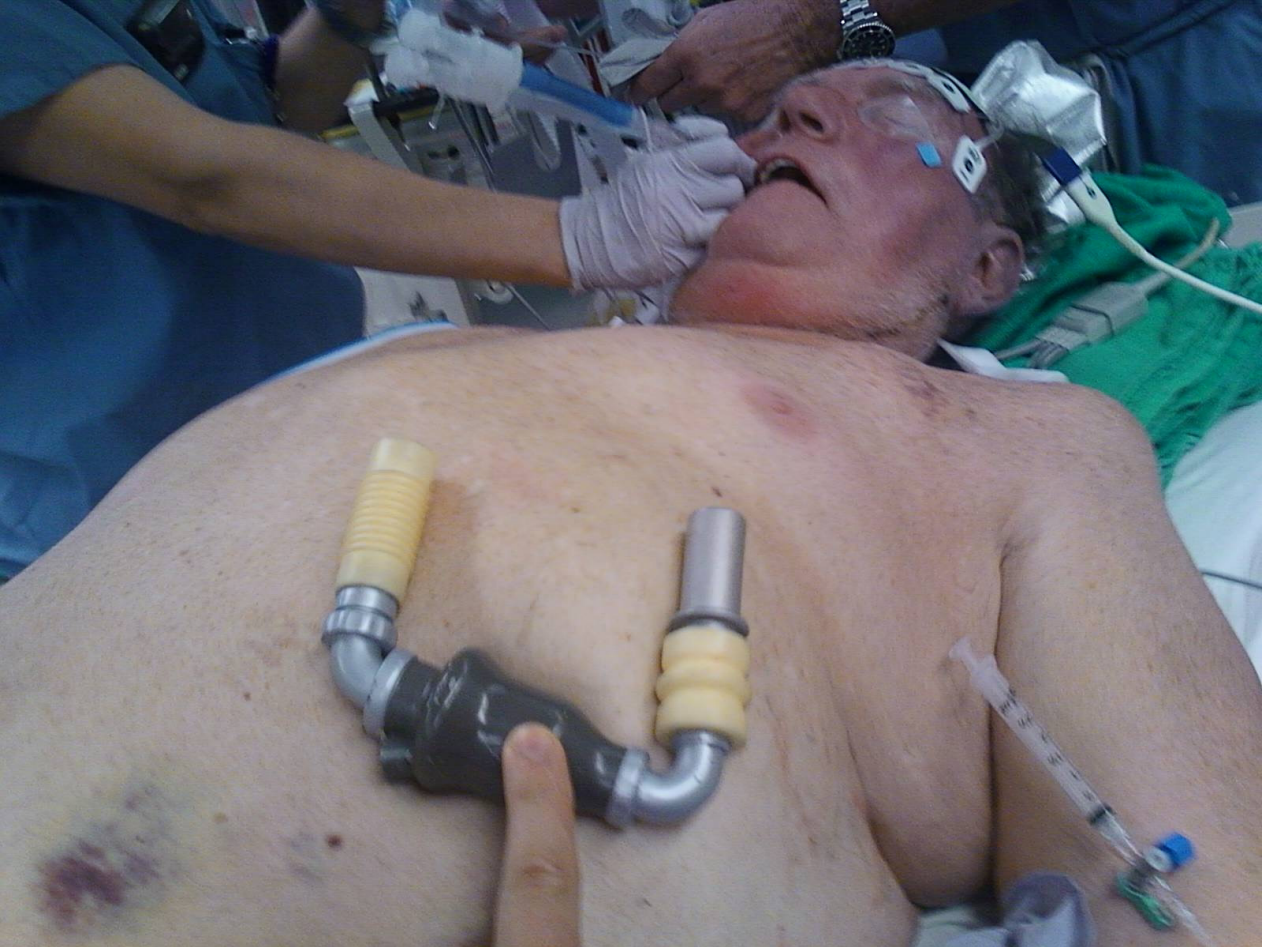
**Preoperative plan with HM-II™ Model**.

In order to avoid the scar tissue related to the prior cardiac surgery, a deliberate procedure was planned to establish an inflow site, outflow site, and LVAD pocket. As such, a mini-upper-sternotomy, a limited left thoracotomy, and a partial midline upper abdominal pre-peritoneal laparotomy was instituted (Figures 1,2). Cardiolpulmonary (CPB) bypass was established with an aortic cannula and a femoral venous cannula. However, CPB could have been established in other ways, including femoral artery and vein, femoral vein and aorta, femoral artery and right atrium (RA), or aorta and right atrium. Our preference was to establish aorta and femoral vein since we could identify enough aorta for direct cannulation and allow for an outflow graft; additionally, femoral venous cannulation allowed us to avoid extending the mini sternotomy in order to expose the RA. In all three cases, three-dimensional CT Scanning of the chest with accompanying CT angiography (including the femoral vessels) was helpful in planning the incisions and cannulation strategy. We could easily determine where to extend (and limit) the upper sternotomy, where to locate the anterolateral thoracotomy over the LV apex, and whether or not the femoral vessels or aorta could be used for CPB. The surgical findings in all three cases were noteworthy and could be described independently: cannulation, inflow, outflow, and LVAD pocket.

#### Cannulation

Since it was our preference to establish CPB using the femoral vein and aorta--understanding that alternatives are perfectly acceptable--we performed a mini upper sternotomy with a hockey-stick incision to the right lateral side. The extent of the incision was based on the CT scan and the goal was to expose the aorta up to its root for cannulation and outflow graft attachment. Femoral venous cannulation was performed in a standard fashion with a guidewire passed into the right heart and confirmed in position by TEE--a suitable size percutaneous venous cannula (e.g. 21 Fr) was positioned accordingly.

#### Inflow

**Figure 3:**
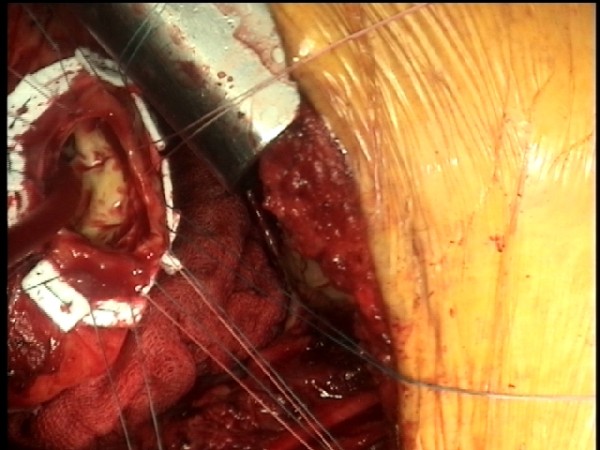
**Intraop Exposure of LV Apex**.

Although absolute identification of the LV apex with the limited exposure was a concern, we were confident that we were in the correct area based on the preoperative CT scan. Although we did not need to remove or deliberately divide a rib, we were prepared to do so if needed. Transesophageal echocardiography (TEE) was essential in confirming the inflow site. Once the thoracotomy incision was made and the ribs spread apart to expose the LV apex (Figure 3), we made a small ventriculotomy and inserted a Foley catheter with a 30 cc balloon and inflated it, watching the entire process with trans-esophageal echocardiography (TEE). In the event that we were in a suboptimal location, we were prepared to close the ventriculotomy with a mattress-pledgetted suture, much like that performed for LV apical vent closure. It should be noted that we were already cannulated for CPB prior to any of these maneuvers.

#### Outflow

**Figure 4:**
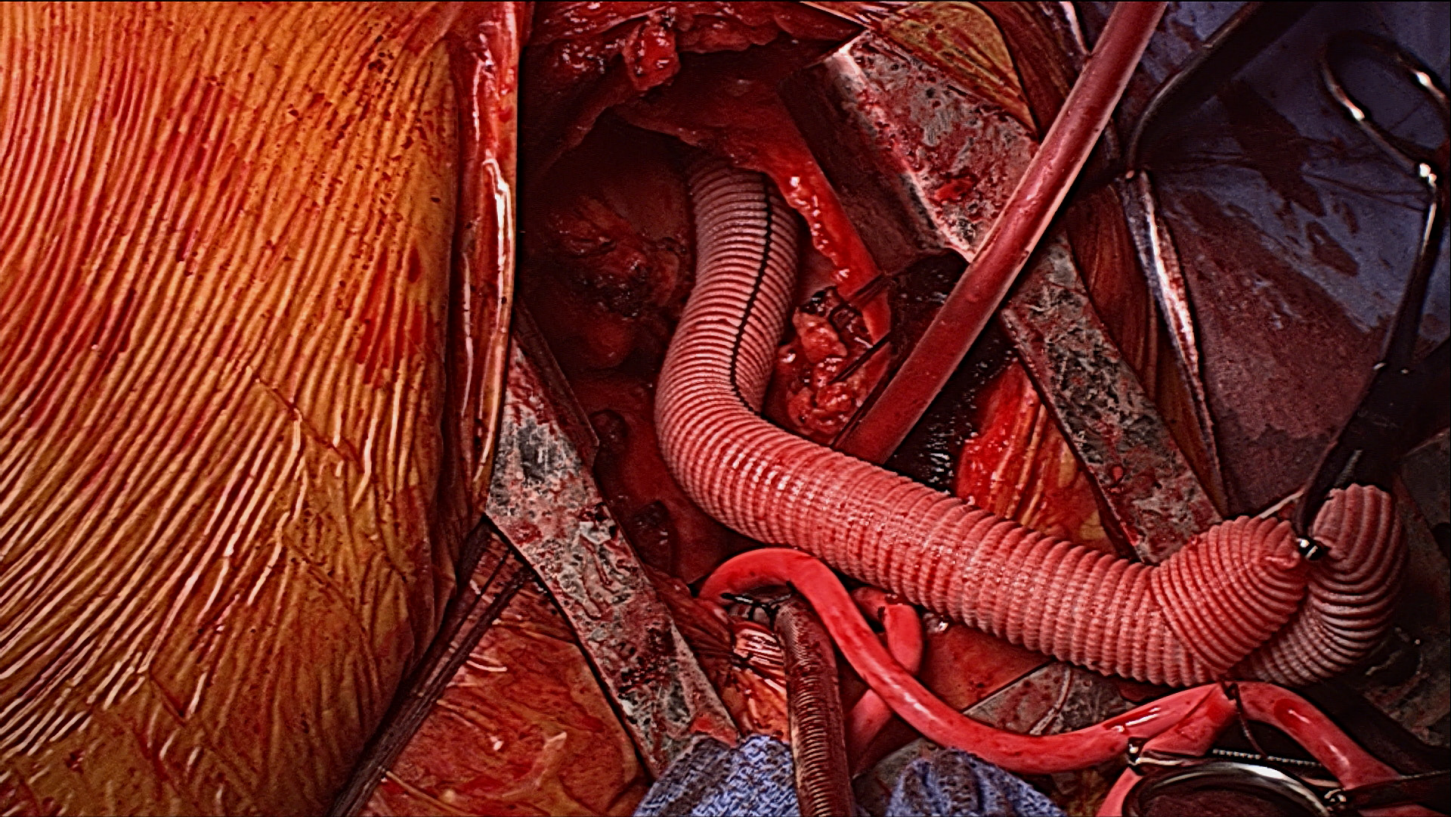
**Intraoperative exposure of Aorta with Outflow Graft**.

The outflow graft was directed toward the anterolateral aspect of the aorta. The hockey-stick incision was helpful in exposing enough aorta to determine cannulation and outflow graft location (Figure 4). However, in the cases in which prior bypass grafts were present, we were prepared to cannulate the femoral artery or right axillary artery to provide more space on the ascending aorta. An opening in the right pleura from the mini-sternotomy and another pleurotomy over the right hemidiaphragm permitted the outflow graft to curve from the LVAD pocket site up to the distal anastamosis site.

#### LVAD Pocket

An upper pre-peritoneal incision was made and the LVAD pocket created in the subrectus muscle plane. Dissection laterally to both sides allowed for direct communication to the inflow site on the patient's left and the outflow pathway in the hemithorax on the patient's right side. The connections were easily established and no distortion or kinking of the LVAD was encountered in the varied body profiles of the patients described.

## Discussion

The use of LVADs for end-stage heart failure patients as a Destination Therapy device is growing in popularity, largely because transplantation numbers are stagnant and medical therapy has done little to change the prognosis of Class IV or Stage D patients who fail to respond to optimal treatment. However, many of the DT patients are older and sicker than their BTT counterparts and often have had prior cardiac surgery and significant co-morbidities. The risk of the LVAD implantation, therefore, is increased and methods to reduce the risk should be instituted. As such, preoperative maneuvers--such as nutritional optimization, improvement in right heart parameters, correction of hematologic abnormalities, and so forth--have translated into less perioperative problems [5,6]. Postoperative maneuvers have similarly resulted into better outcomes as a more formal critical care approach to the complexities of all the organ systems is addressed [7,8]. Intraoperatively, any maneuver that can minimize trauma and avoid complication--bleeding or otherwise--is worth exploring. Thus, the alternative technique described above accomplishes the goal of implanting the LVAD without exposing the entire heart. A similar approach was described by Gregoric et al as well as Anyanwu in which a right thoracotomy and left subcostal incision were utilized [9,10]. Recently, Schmitto et al described placement of an implantable centrifugal pump using an upper hemisternotomy and anterolateral thoracotomy [11]. These authors found this approach to be less invasive and less traumatic in terms of potential for intraoperative catastrophes and postoperative bleeding. We found our approach to provide similar advantages and have now adopted our technique for all implants, high risk and otherwise.

The favorable features of the alternative technique described as well as those referenced are several. For example, the LV apex in end-stage heart failure patients is so laterally displaced that a true midline approach requires an extensive lateral dissection for proper orientation of the inflow cannula. On the contrary, the limited lateral thoracotomy is directly over the LV apex making the inflow connection far easier. In addition, the traditional trans-sternal approach requires extensive dissection through scar tissue in redo cases, thereby putting into jeopardy previously placed bypass grafts; the scar tissue dissection alone can result in excessive bleeding even in the absence of previous grafts. Furthermore, inadvertent entry into the right ventricle or other cardiac chamber can be disastrous. These aspects were of particular concern in the first case described. We deliberately wanted to avoid the dense adhesions of the CorCap™, which is an artificial material that fixes firmly to the heart and surrounding tissues. The hazards of reoperating on a patient with a previously placed CorCap™ was well described by Schroder and colleagues during the cardiectomy of the recipient at the time of transplantation [12].

In summary, we describe an alternative technique of LVAD implantation in high risk DT patients that minimizes the potential for intraoperative complications. The cases themselves are illustrative of this point, however, the high risk nature of the procedure is still readily apparent. Although we avoided potential intraoperative problems, the "high-risk" nature of the case shifted more toward the patient profile than the operative technique. This was poignantly presented by Vitale and others in their manuscript entitled "A call for guidance in the use of left ventricular assist devices in older adults" [13]. In retrospect, perhaps the lesson of these cases is to apply the technique to a less morbid population where the benefit of the surgical modification may be better appreciated.

## Consent

This retrospective review was approved by the Investigational Review Board of the Lankenau Medical Center in Wynnewood, PA.
